# Significance of the Entire Appendiceal Evaluation in the Diagnosis of Serrated Lesions, Low-Grade Appendiceal Mucinous Neoplasm, and Appendiceal Diverticulosis Disease

**DOI:** 10.3389/fonc.2021.812794

**Published:** 2022-01-18

**Authors:** Fang Li, Yiyan Lu, Fang Hou, Ruiqing Ma, Dezhong Wang, Changhai Qi

**Affiliations:** ^1^ Department of Pathology, Aerospace Center Hospital, Beijing, China; ^2^ Department of Myxoma, Aerospace Center Hospital, Beijing, China; ^3^ Department of General Surgery, Aerospace Center Hospital, Beijing, China

**Keywords:** appendix, diagnosis, serrated polyp, low-grade appendiceal mucinous neoplasm, diverticular disease

## Abstract

**Objective:**

This study was conducted in order to investigate the significance of the entire appendiceal evaluation in the pathological diagnosis of appendiceal serrated lesions, low-grade appendiceal mucinous neoplasm (LAMN), and appendiceal diverticulosis disease (ADD).

**Methods:**

A total of 702 appendectomy specimens diagnosed from 2017 to 2020 were reviewed retrospectively. The specimens were divided into two groups according to the different sampling procedures. In group 1, the vast majority of 337 specimens were partially submitted by routine sampling within 18 months from October 2017 to March 2019. In group 2, 365 of specimens were entirely submitted and examined within 18 months from April 2019 to October 2020. The incidence and pathological features of serrated lesions, LAMN, and ADD in the two groups were compared and analyzed. The clinicopathological characteristics between different entities were also studied.

**Results:**

Forty appendiceal serrated lesions, 8 LAMNs, and 21 diverticula were accidentally detected in 702 appendectomy specimens. As compared with group 1, the incidence of appendiceal serrated lesions in group 2 was significantly increased (9.3% vs. 1.8%, *P *< 0.01), especially for the serrated lesions without dysplasia (7.4% vs. 1.2%, *P *< 0.01). The entire sampling revealed that loss of lamina propria and replacement with dysplastic mucinous epithelium were statistically significantly associated with LAMN rather than serrated lesions and ADD (*P *< 0.01 and *P *< 0.01, respectively). Mural mucin deposition and fibrosis were useful features to distinguish LAMN from simple serrated lesions (*P *< 0.01 and *P *< 0.05, respectively), but mucin deposition was useless for the distinction between LAMN and ADD (*P* > 0.05) or serrated lesions combined with ADD.

**Conclusion:**

Our study highlights the importance and necessity of careful gross assessment and histologic examination of the entire appendectomy specimen, since the association with unexpected appendiceal lesions is significant and cannot be ignored. The entirely submitted appendix is more sensitive for the detection of appendiceal serrated lesions. In addition, thorough examination and evaluation are essential to distinguish the key pathological features between appendiceal serrated lesions, LAMN, and ADD.

## Introduction

The appendix is a common specimen in the surgical pathology laboratory. Simple appendectomy is often performed because of clinical suspicion of acute appendicitis. Surgical procedures for tumors and inflammation of the right colon and ovarian tumors usually involve the appendix. If there are no obvious gross findings other than acute appendicitis, routine appendix sampling is usually performed. In cases with suspicious lesions in clinical and gross examination, additional sections may be required. Moreover, when suspicious findings are observed in subsequent microscopic examination, the remaining appendix specimens should be entirely submitted and evaluated to identify the nature of the lesion. If a neoplastic appendiceal lesion is confirmed, the resection margin should also be evaluated.

Serrated lesions are a group of heterogeneous epithelial lesions with serrated structure. According to the recently published fifth edition of the 2019 World Health Organization (WHO) Classification of Tumors of the Digestive System, appendiceal serrated lesions are classified into three subtypes, namely, hyperplastic polyp (HP), serrated lesion without dysplasia (SL), and serrated lesion with dysplasia (SLD) (1). Previous studies have revealed that appendiceal serrated lesions are usually detected incidentally in the appendix removed for appendicitis and other diseases (2, 3). Furthermore, diverticula and the early stage of low-grade appendiceal mucinous neoplasms (LAMNs) may also be presented with symptoms of acute appendicitis, but these lesions are different entities and may be difficult to distinguish from each other.

For a while, we have detected some incidental appendiceal serrated lesions, even acellular mucin with involvement of subserosa. In those cases, the remaining appendices should be entirely submitted for evaluation. It may be difficult to identify the resection margins of the processed appendix specimens. Therefore, a time point was set after which all appendectomy specimens were entirely submitted for examination. The aim of this study was to reveal the incidence and histopathological characteristics of incidental appendiceal serrated lesions, LAMN, and appendiceal diverticulosis disease (ADD) between the two different sampling procedures in the same period. The clinicopathological features between different entities were also compared and analyzed.

## Materials and Methods

### Patients

A total of 702 appendectomy specimens from October 2017 to October 2020 treated at the Aerospace Center Hospital (Beijing, China) due to appendicitis, other inflammatory diseases, and non-appendiceal tumors were selected. The inclusion criteria were as follows: appendectomy specimens due to clinical diagnosis of appendicitis, right colon inflammation, and non-appendiceal tumors including the right colon, female reproductive system, etc. The exclusion criteria were as follows: patients with appendiceal tumors and peritoneal pseudomyxoma were clinically diagnosed before operation.

### Sample Methods

For group 1, 337 cases were collected from October 2017 to March 2019. The overwhelming majority of the appendiceal specimens were partially submitted by routine sampling. If there were no grossly obvious changes other than acute appendicitis, three sections were submitted for histological evaluation, namely, two representative transverse sections of the base and body and a longitudinal section of the tip. A total of 3.3% (11/337) of cases were either completely submitted due to suspicious macroscopic observations or the remaining specimens were resampled due to microscopic findings, including four appendiceal serrated lesions, three LAMNs, and four diverticula. For group 2, 365 cases were collected from April 2019 to October 2020. The entire appendix was submitted for evaluation, even if the appearance is normal. The sampling process used was to cut the base and body of the appendix with thin transverse sections and to split the distal 2-cm tip of the appendix with longitudinal sections. All transverse sections of the margin, base, and body and longitudinal sections of the tip were placed into tissue cassettes in a sequential fashion. Formalin-fixed, paraffin-embedded sections were stained with hematoxylin and eosin for routine histological evaluation. The corresponding glass slides were retrieved.

### Diagnosis Criteria

According to the 2019 WHO criteria and Peritoneal Surface Oncology Group International (PSOGI) consensus (1, 4), cases were reviewed by two experienced gastrointestinal pathologists. The appendiceal serrated lesions were classified into three types, namely, HP, SL, and SLD. HP and SL shared the same features of epithelial serration without cytologic dysplasia. HP showed serration limited to their luminal aspects. In SL, the mucosa demonstrated distorted crypts with serration and crypt dilatation extending to the base of the crypts. In SLD, the serrated architecture was maintained. The dysplasia could resemble routine adenoma-like dysplasia, serrated dysplasia, or traditional serrated adenoma-like dysplasia, and multiple morphological patterns of dysplasia might be observed within a single polyp (5). The dysplasia were classified as low-grade or high-grade according to cellular and structural dysplasia. LAMNs were associated with the replacement of the appendiceal mucosa with a villous, undulating, or flattened neoplastic mucinous epithelium that demonstrated low-grade cytologic dysplasia. The submucosa and muscularis propria could show varying degrees of fibrosis and hyalinization. Pushing invasion with a broad front of mucinous epithelium that expands into the mural wall without destructive features was also a common feature of LAMN. The mucin could dissect through the structures of the appendix and extended to the peritoneal surface or cause rupture of the appendix. ADD was characterized by herniation of appendiceal mucosa through microanatomical defects in the muscularis propria, and there was no evidence of neoplastic epithelium and pushing infiltration.

### Study Parameters

Clinical information about age, gender, and clinical manifestations was obtained from the medical records. Data regarding appendiceal diameter and presence and location of grossly visible cysts and/or mucin were collected by reviewing the pathological archives. The following histopathological parameters were evaluated and compared between the different submission groups and/or disease entities, including lesion localization, number, length, margin, mucosa structure (retention/loss of lamina propria, serration, dysplastic serrated epithelium, dysplastic mucinous epithelium, atrophy), mural structure (mucin deposits, fibrosis, calcification), and other alterations (complicated tumors, appendicitis, diverticulitis).

### Statistical Analysis

Statistical analysis was conducted using GraphPad Prism 8.0.2. Two-tailed chi-square test, continuous correction chi-square test, and Fisher’s exact test were used for comparative analysis between groups. A *P*-value <0.05 was considered statistically significant.

## Results

### Clinical Features

The clinical features between the two submission groups are summarized in **Table 1**. Three hundred and thirty-seven appendectomy specimens were collected in group 1, consisting of 162 males and 175 females, with a male to female ratio of 1:1.08. The age ranged from 8 to 98 years, with a median age of 43 years. In group 2, 365 specimens of appendix were entirely submitted and examined, consisting of 184 males and 181 females, with a male to female ratio of 1.02:1. The age ranged from 8 to 89 years, and the median age was 47 years. There were 517/702 (73.6%) of patients in the two groups who presented with appendicitis and underwent simple appendectomy. Acute appendicitis accounted for 70.0% (236/337) and 66.6% (243/365), and chronic appendicitis accounted for 8.0% (27/337) and 3.0% (11/365), respectively, in groups 1 and 2. A total of 168/702 (23.9%) of non-appendiceal neoplasms underwent tumor resection or radical surgery, including digestive tumors, female reproductive tumors, and other tumors. Other acute abdominal pain was performed by exploratory laparotomy in a few cases (15/702).

**Table 1:** Clinical features between different appendix submission groups.

	Group 1 (*N* = 337)	Group 2 (*N* = 365)	Total number (*N* = 702)
Gender			
Male	162 (48.1%)	184 (50.4%)	346 (49.3%)
Female	175 (51.9%)	181 (49.6%)	356 (50.7%)
Median age (range) (years)	43 (8–98)	47 (8–89)	
Clinical diagnosis			
Acute appendicitis	236 (70.0%)	243 (66.6%)	479 (68.2%)
Chronic appendicitis	27 (8.0%)	11 (3.0%)	38 (5.4%)
Mucoceles	0	2 (0.5%)	2 (0.3%)
Digestive neoplasms	41 (12.2%)	76 (20.8%)	117 (16.7%)
Female reproductive neoplasms	23 (6.8%)	18 (4.9%)	41 (5.8%)
Other neoplasms	5 (1.5%)	5 (1.4%)	10 (1.4%)
Other acute abdominal pain	5 (1.5%)	10 (2.7%)	15 (2.1%)

The clinical findings among patients with appendiceal serrated lesions, LAMN, and ADD are demonstrated in **Table 2**. A total of 40 appendiceal serrated lesions, 8 LAMNs, and 21 diverticula were found in the two different submission groups. The incidence of accidental serrated lesions (5.7%) and ADD (3.0%) was higher than LAMN (1.1%). Gender and age differences were not statistically significant between entities. Patients with ADD were more likely to present with symptoms related to acute appendicitis than those with LAMN (57.1% vs. 12.5%, *P *< 0.05). Appendiceal serrated lesions were more likely associated with other neoplasms as compared with ADD (50% vs. 4.8%, *P *< 0.01).

**Table 2:** Clinical and gross features among patients with appendiceal serrated lesions, LAMN, and ADD.

Features	Serrated lesions (*N* = 40)	LAMN (*N* = 8)	ADD (*N* = 21)	*P*-value^a^	*P*-value^b^
Gender				0.3645	0.0957
Male	20 (50%)	2 (25%)	13 (61.9%)		
Female	20 (50%)	6 (75%)	7 (33.3%)		
Mean age (years)	65	53	52		
Clinical diagnosis					
Acute appendicitis	18 (45%)	1 (12.5%)	12 (57.1%)	0.1868	<0.05
Chronic appendicitis	2 (5%)	1 (12.5%)	2 (9.5%)	–	–
Mucoceles	0	1 (12.5%)	1 (4.8%)	–	–
Neoplasms	20 (50%)	3 (37.5%)	1 (4.8%)	0.7961	0.525
Other acute abdominal disease	0	2 (25%)	1 (4.8%)	–	–
Gross features					
Appendicitis	18 (45%)	1 (12.5%)	8 (38%)	0.1868	0.3715
Diameter range (mean diameter)	0.4–2 cm (0.9 cm)	1–6 cm (2.4 cm)	0.7–2 cm (1.1 cm)	<0.01	0.1098
Diffuse dilation	0	4 (50%)	0	<0.01	<0.01
Localized protrusion	2 (5%)	1 (12.5%)	3 (14.3%)	0.4288	>0.9999
Luminal mucin	0	5 (62.5%)	1 (4.8%)	<0.01	<0.01
Extramural mucin	0	0	1 (4.8%)	–	–

a P-value, serrated lesions vs. LAMN.

b P-value, LAMN vs. ADD.

### Incidence of Lesions

The incidence of the serrated lesions, LAMN, and ADD between different appendix submission groups are demonstrated in **Table 3**. The incidence of accidental lesions detected in group 2 (13.4%, 49/365) was statistically higher than that in group 1 (4.7%, 16/337) (*P *< 0.01). The incidence of appendiceal serrated lesions in group 2 (9.3%, 34/365) was statistically higher than that in group 1 (1.8%, 6/337) (*P *< 0.01). The incidence of appendiceal SLs in group 2 (6.8%, 25/365) was significantly higher than that in group 1 (1.2%, 4/337) (*P *< 0.01). The incidence of appendiceal HPs and SLDs in group 2 was slightly higher than that in group 1, but the difference was not statistically significant (*P* > 0.05).

**Table 3:** The incidence of appendiceal serrated lesions, LAMN, and ADD between different appendix submission groups.

Entities	Group 1 (*N* = 337)	Group 2 (*N* = 365)	*P*-value
Detected lesions	16 (4.7%)	49 (13.4%)^a^	<0.01
Serrated lesions	6 (1.8%)	34 (9.3%)	<0.01
HP	1 (0.3%)	2 (0.6%)	0.6102
SL	4 (1.2%)	25 (6.8%)	<0.01
SLD	1 (0.3%)	7 (1.9%)	0.0958
LAMN	3 (0.9%)	5 (1.4%)	0.8085
ADD	7 (2.1%)	14 (3.8%)	0.1718

a Cases included four serrated lesions combined with diverticula.

The incidence of LAMNs and diverticula in group 2 was slightly higher than that in group 1 (1.4% vs. 0.9%, 3.8% vs. 2.1%, respectively) without statistically significant difference (*P* > 0.05).

### Histopathological Features

Five out of 8 (62.5%) LAMNs had grossly obvious abnormalities, indicating the presence of a neoplasm. Fifty percent (4/8) of cases were associated with diffuse appendiceal dilatation, but there were no cases of serrated lesions and ADD (*P *< 0.01 and *P *< 0.01, respectively). The degree of appendiceal dilatation was also greater among LAMNs than serrated lesions (mean: 2.4 vs. 0.9 cm, *P *< 0.01). Five out of 8 (62.5%) LAMNs contained luminal mucus, whereas none of the serrated lesions featured visible luminal mucin (*P *< 0.01) and only 4.8% of appendiceal diverticula (*P *< 0.01). In contrast, serrated lesions and appendiceal diverticula tended to display gross changes of appendicitis. Eighteen (45%) serrated lesions and 8 (38%) diverticula featured grayish brown and rough serosa, purulent moss, and edema compared with only 1 (12.5%) LAMN, but the difference was not statistically significant (*P* > 0.05).

**Figure 1:**
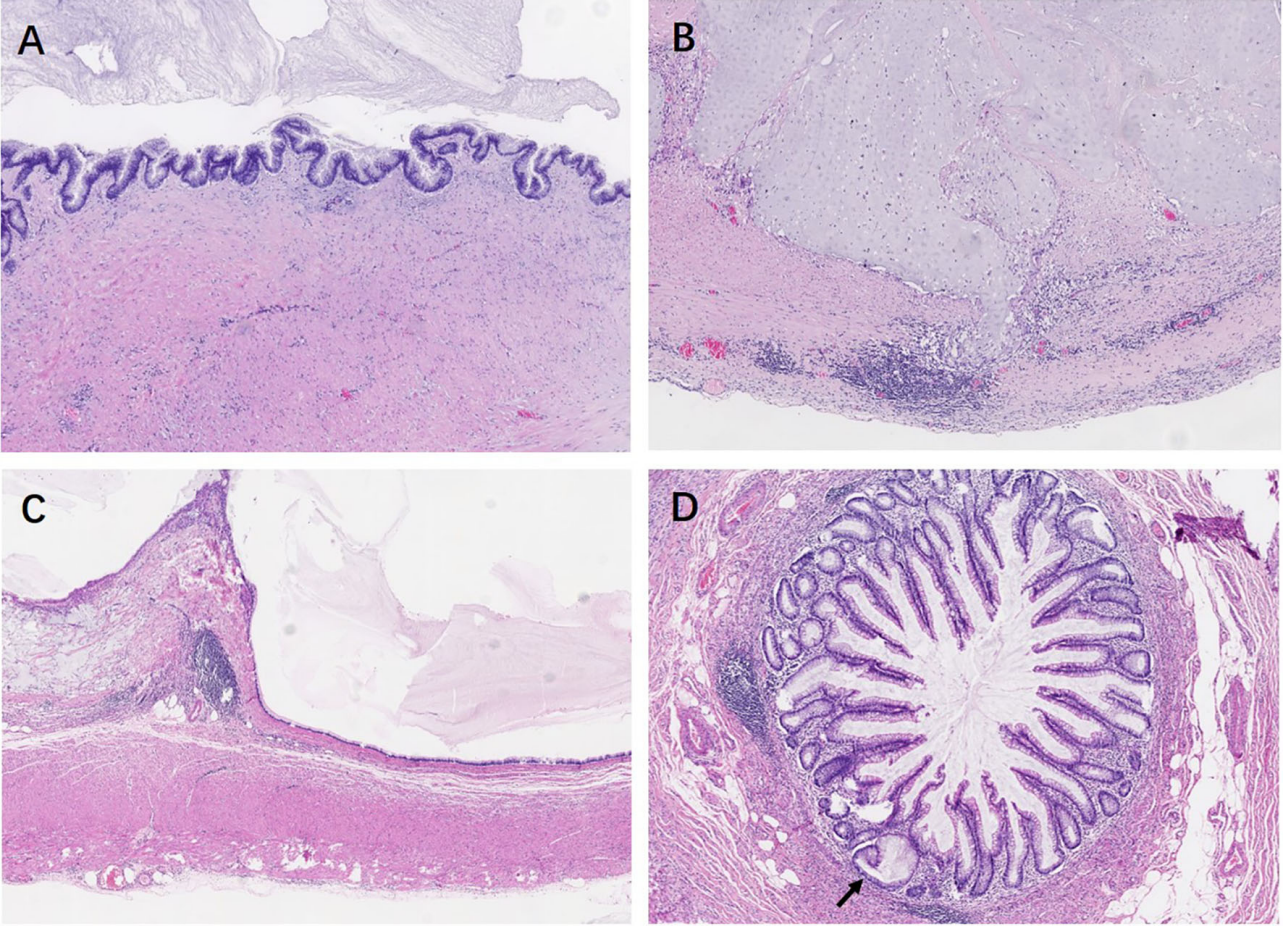
Low-grade appendiceal mucinous neoplasms. **(A)** LAMN is characterized by loss of the lamina propria and muscularis mucosae and fibrosis of the submucosa. The mucosa is replaced by an undulating neoplastic mucinous epithelium that demonstrates low-grade cytologic dysplasia. **(B)** By thorough sampling and evaluation, this case is confirmed as pT3 stage with subserosa involvement of acellular mucin. **(C)** The neoplastic mucinous epithelium has a flattened architectural arrangement. Note the dissected mucin in the wall on the left. **(D)** Other section without lumen dilation shows the serrated lesions with L-shaped serrations in the basal crypt (black arrow).

Pathological features between different entities in the entirely submitted appendices are summarized in **Table 4**. In the entire appendix assessment, loss of lamina propria (*P *< 0.01) and replacement with dysplastic mucinous epithelium (*P *< 0.01) were significantly associated with LAMN rather than serrated lesions and ADD. Mural mucin deposition and fibrosis were useful features to distinguish LAMN from simple serrated lesions (*P *< 0.01 and *P *< 0.05, respectively), but mucin deposition was useless for the distinction between LAMN and ADD (*P* > 0.05) or serrated lesions combined with ADD. Neoplastic mucinous epithelium demonstrated low-grade cytologic dysplasia with a villous, undulating, and flattened architectural arrangement (**Figures 1A, C** **)**. Thorough sampling was also helpful to display fibrosis, calcifications, stage (**Figure 1B**), and margins. Three (37.5%) LAMNs were found to have serrated lesions in the background, especially in the mucosal area without dilation (**Figure 1D**).

**Table 4:** Pathological features of appendiceal serrated lesions, ADD, and LAMN in the entirely submitted appendices.

Features	Serrated lesions^a^ (*N* = 34)	ADD^b^ (*N* = 14)	Serrated lesions combined with ADD (*N* = 4)	LAMN (*N* = 8)	*P*-value^c^	*P*-value^d^
Mucosal structure						
Retention of lamina propria	34 (100%)	13 (92.9%)	4 (100%)	0	<0.01	<0.01
Serrated epithelium without dysplasia	28 (82.4%)	0	3 (75%)	3 (37.5%)	<0.05	<0.05
Dysplastic serrated epithelium	6 (17.6%)	0	1 (25%)	0	0.4704	–
Dysplastic mucinous epithelium	0	0	0	8 (100%)	<0.01	<0.01
Atrophy	3 (8.8%)	12 (85.7%)	4 (100%)	0	–	<0.01
Mural structure						
Mucin deposition	0	9 (64.3%)	4 (100%)	5 (62.5%)	<0.01	0.9999
Fibrosis	0	0	0	2 (25%)	<0.05	0.1212

a Cases of serrated lesions included 4/6 of serrated lesions in group 1 and 30/34 of simple serrated lesions in group 2.

b Cases of ADD included 4/7 of ADD in group 1 and 10/14 of simple ADD in group 2.

c P-value, serrated lesions vs. LAMN.

d P-value, LAMN vs. ADD.

**Figure 2:**
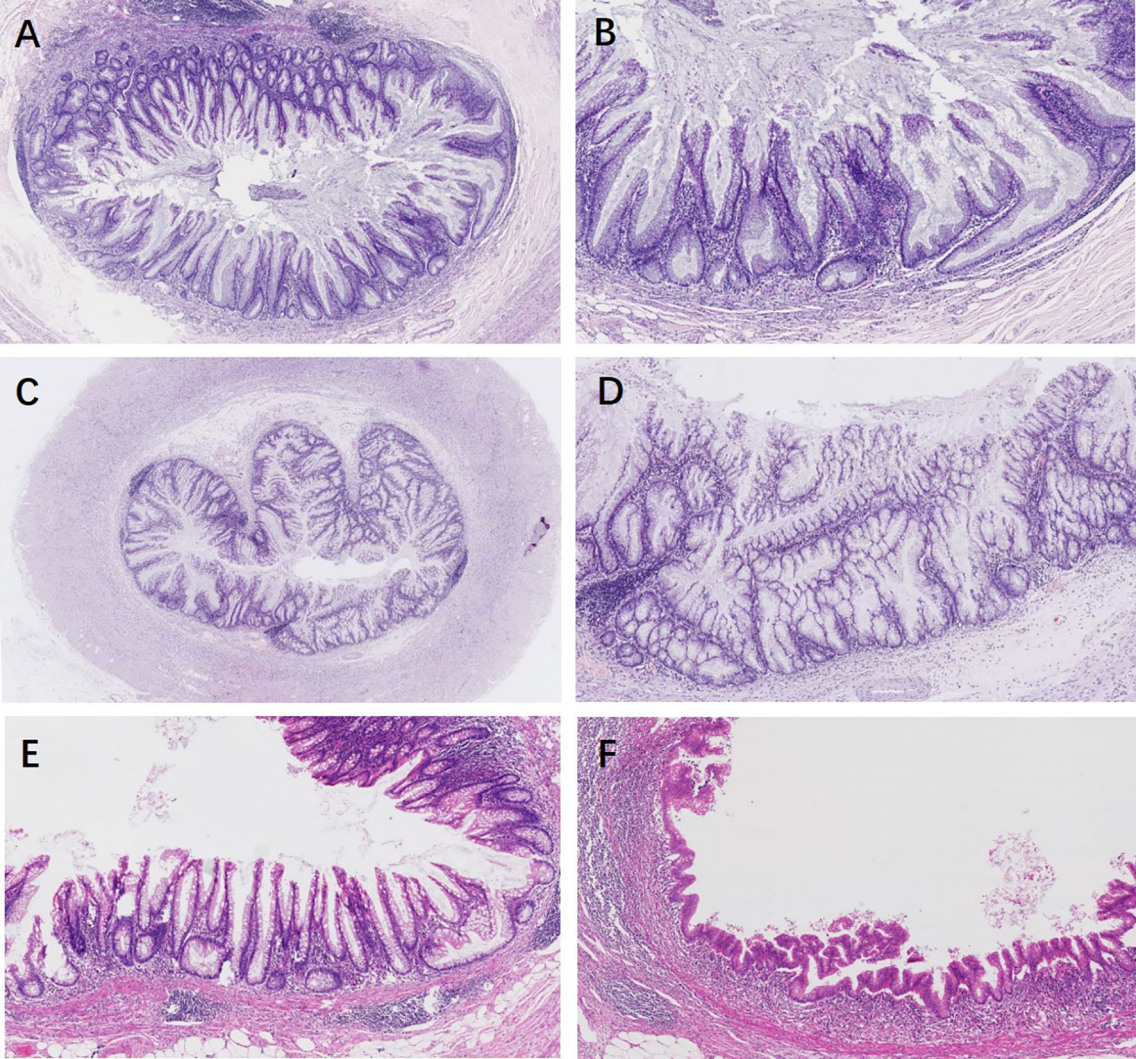
Appendiceal serrated lesions. **(A, B)** Serrated lesions without dysplasia. **(A)** Serrated lesions without dysplasia involve a portion of the appendiceal circumference. **(B)** On higher power, serration and dilation extend to the crypt bases with abnormal L shapes. **(C–F)** Serrated lesions with dysplasia. **(C)** Serrated lesions with dysplasia involve the entire appendiceal circumference. **(D)** On higher power, the dysplasia is low grade, resembling traditional serrated adenoma-like dysplasia. Multiple ectopic crypt formations and villous growth are noted. **(E)** A typical appendiceal serrated lesion with focal area of flattened or scalloped monolayer of epithelium mimic a low-grade appendiceal neoplasm, but maintained an intact lamina propria and muscularis mucosae **(F)**.

**Figure 3:**
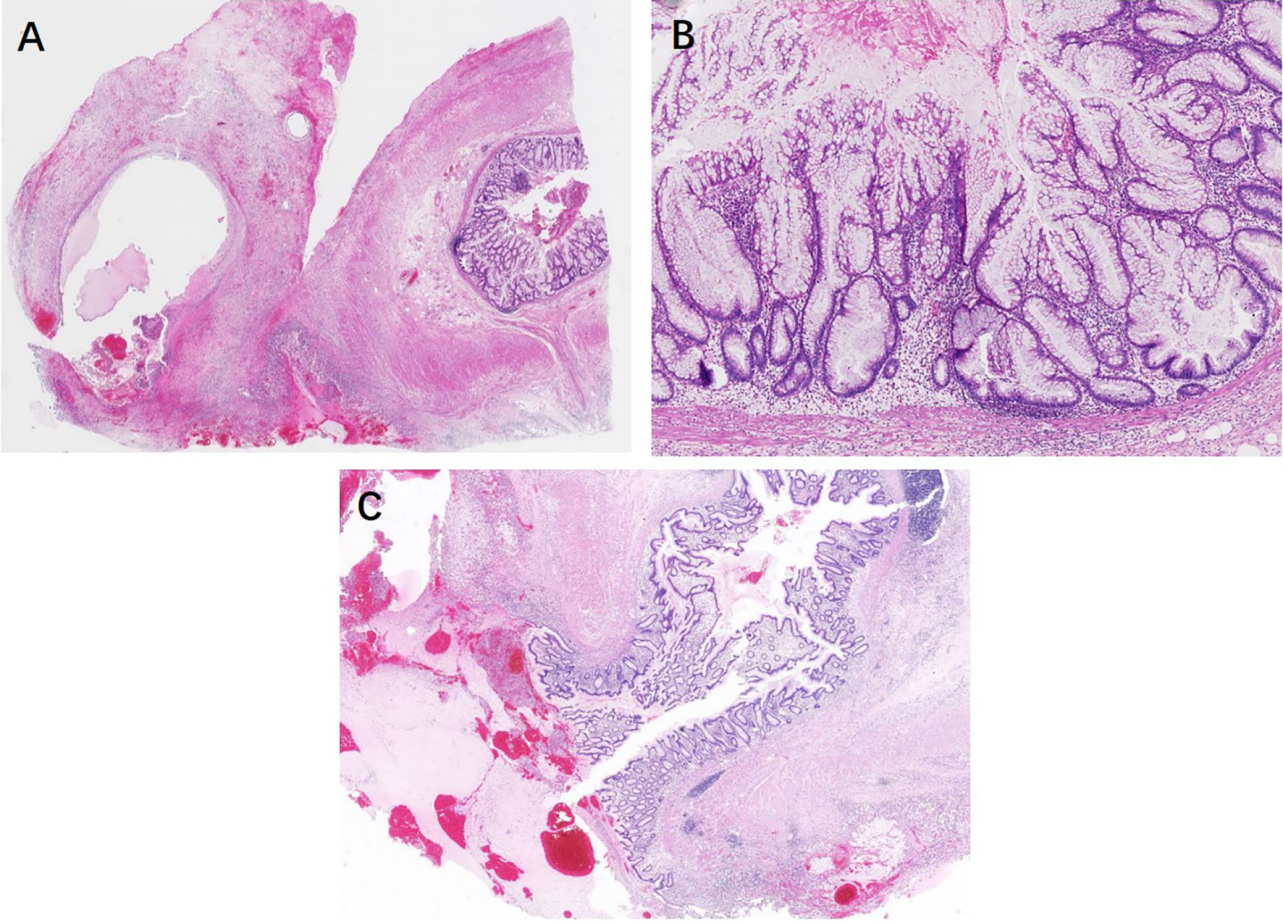
Serrated lesions with dysplasia coexistence of ruptured diverticula. **(A)** The mucus cyst is apparently found within the mesoappendix at the tip. **(B)** On higher power, the mucosa is lined with distorted serrated crypts with low-grade dysplasia. **(C)** On the other sections of the entirely submitted appendix, the ruptured diverticulum lined with a relatively normal mucosa rules out a low-grade appendiceal mucinous neoplasm.

Appendiceal HPs showed serration limited to the luminal aspects. The mucosa of SLs demonstrated abnormal crypt proliferation with elongated and serrated crypt profiles. Serration and dilation extended to the crypt bases with abnormal shapes including L shapes and inverted T shapes (**Figures 2A, B** **)**. Cytological dysplasia was not found in all cases of appendiceal HP and SL. Four SLDs showed low-grade dysplasia, similar to traditional serrated adenoma-like dysplasia, with multiple ectopic crypt formations and villous growth (**Figures 2C, D** **)**. Three SLDs showed serrated architecture with focal flattened or scalloped monolayer of epithelium, but maintained the intact lamina propria and muscularis mucosae (**Figures 2E, F** **)**. There were no significant differences in terms of lesion localization, length, and resection margins between the two sampling groups (*P* > 0.05). However, 87.5% (35/40) of appendiceal serrated lesions had a local continuous distribution, usually involving the tip of the appendix (45%, 18/40). Seventeen out of 40 (42.5%) lesions were less than 6 mm in length. In group 2, four serrated lesions combined with ADD, either in both intraluminal and involving the diverticulum/a (three cases) or in the lumen away from the diverticulum (one case) (**Figure 3**).

**Figure 4:**
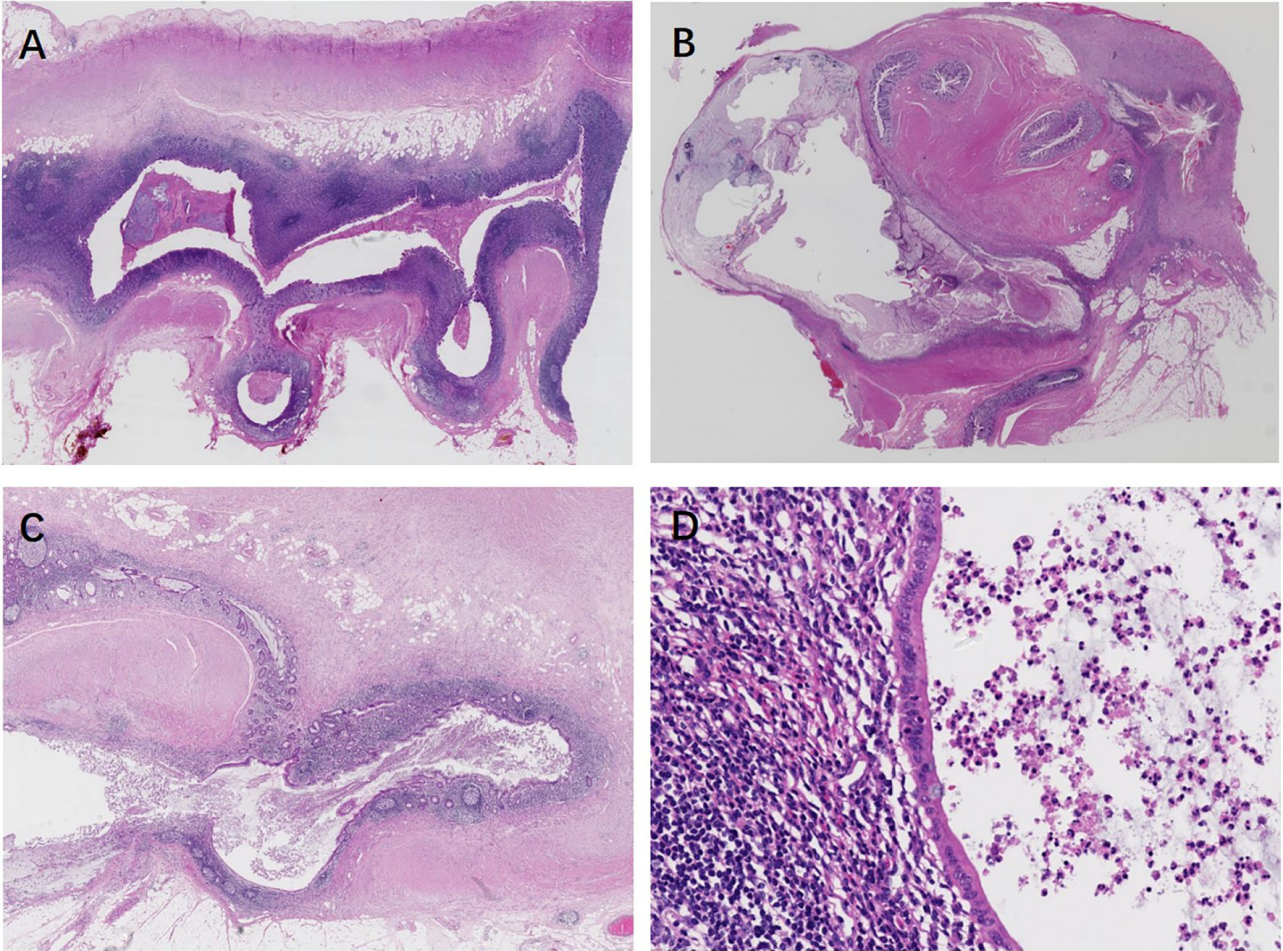
Appendiceal diverticulosis disease. **(A)** Multiple intact diverticula herniate through the muscularis propria. **(B–D)** Ruptured appendiceal diverticula. **(B)** Multiple ruptured diverticula lead to tip deformation and subserosal mucus cyst formation. **(C)** A ruptured appendiceal diverticulum is lined with mucosa preserved of the relatively normal lamina propria, but there is an epithelial monolayer in the area below the picture. Mucin deposit in the mesoappendix. **(D)** At higher magnification, atrophy mucosa lined with a monolayer epithelium shows a lack of mucin and nuclear abnormalities, and there are abundant lymphoid follicles in the underlying lamina propria.

There were no significant differences in grossly visible findings, number of diverticula, localization, mucosa changes, extramural mucin, and combined lesions in ADD among different sampling groups (*P* > 0.05). However, 81% (17/21) of diverticula most commonly involved the distal appendix, and there was a tendency for multiple locations (57.1%, 12/21) (**Figures 4A, B** **)**. Another common feature of ADD was mucosa atrophy (76.2%, 16/21), lined with a monolayer of epithelium without dysplasia (**Figures 4C, D** **)**. In addition, 61.9% (13/21) of the cases were found to have extramural mucin in gross examination and/or subsequent microscope scanning. Fourteen out of 21 (66.7%) cases had coexistence acute inflammation, including acute diverticulitis and acute appendicitis, consistent with clinical symptoms.

## Discussion

Great progress has been made in the diagnostic criteria and molecular manifestations of serrated lesions of the colorectum. The terminology proposed by the fifth edition of the 2019 WHO has been changed from the previous edition. It is named “colorectal serrated lesions and polyps,” which is classified into five categories, namely, HP, SL, SLD, traditional serrated adenomas (TSA), and unclassified serrated adenomas (6). Previous studies have suggested that KRAS may be more biologically important in the appendix than BRAF, and the serrated carcinogenic pathway may have less relevance in the appendix than in the colon (7). Therefore, the 2019 WHO classification of serrated lesions and polyps in the appendix refers to colorectal counterparts, but they are not completely consistent. HP and SL are morphologically similar to the corresponding colorectal lesions, but TSA is not an independent subtype and is classified into SLD.

The incidence of appendiceal serrated lesions is still unknown. Several reasons may be responsible for it. First, as demonstrated in our study, all the lesions were accidentally found in appendices removed for other reasons, either for appendicitis (50%) or non-appendiceal tumors (50%). Appendiceal serrated lesions without acute appendicitis would not cause clinical symptoms. Colonoscopy is not sensitive to detect the lesions, and there is a lack of specific serological biomarkers. In addition, our study showed that 92.5% (37/40) of serrated lesions had negative gross manifestations except for acute appendicitis. Moreover, nearly half of the serrated lesions were less than 6 mm in length. In routine practice, appendix specimens were partially submitted and only three representative slides were used for evaluation, which may lead to missed diagnosis of small serrated lesions. This fact had been confirmed by comparing with the partial submission group, and the incidence of serrated lesions in completely submitted appendices was significantly increased (9.3% vs. 1.8%), especially SLs (7.4% vs. 1.2%). Renshaw et al. also confirmed that in 100 cases of acute appendicitis, the incidence of sessile serrated adenoma in the appendix of the entire submission was much higher than that of the partial submission (20% vs. 2%), and the lesions were usually less than or equal to three cross sections (8). Yuyucu Karabulut et al. retrospectively analyzed 960 appendectomy specimens. Seventy-one (7.39%) serrated lesions were detected by routine sampling, consisting of 36 HPs (50.7%), 33 SLs (46.48%), and 2 TSAs (2.81%), of which 66 cases were diagnosed with acute appendicitis pre-operation (9). All serrated lesions were found under the microscope, except for one case with positive gross changes, suggesting that extensive sampling is crucial for the identification of appendiceal serrated lesions (9).

The importance of colorectal serrated lesions has increased in the last decade because this type of lesions accounts for about 15%–30% of colorectal carcinomas and follows the serrated pathway of colorectal carcinogenesis (10). This pathway is characterized by an epigenetic mechanism that involves abnormal methylation of CpG islands in the promoter regions of tumor suppressor genes and may be associated with mutations of the BRAF oncogene (10). Mutations in this gene play an equivalent role as KRAS mutations in chromosomal instability colorectal cancer (11, 12). However, it is debated whether serrated lesions of the appendix are associated with the same mutations and risk for malignancy as colonic serrated lesions. Some studies have shown that the pathway differs in appendiceal lesions in that the KRAS mutation is present but the BRAF mutation is less commonly found (7). Interestingly, several studies have revealed that the LAMNs are more likely to be associated with high-frequency mutation of KRAS (13–15). In the present study, we found four incidental LAMNs with a background of serrated lesions. These results indicated an intimate association between appendiceal serrated lesions and LAMN, but detection of a series of serrated lesions and a large number of studies are necessary for confirmation.

Currently, there is still no available surveillance recommended by the guidelines. There are some points worthy to be considered. First, the appendix is part of the colon. If a serrated lesion is detected in the appendix, whether there is a possibility of synchronous or metachronous serrated lesions in the large intestine is unknown. Post-appendectomy surveillance may refer to the recommendations of the colonoscopy guidelines. For instance, according to the 2020 European Society of Gastrointestinal Endoscopy (ESGE) guideline (16), patients with complete removal of any serrated polyp <10 mm without dysplasia do not require endoscopic surveillance and should be returned to screening. If organized screening is not available, repetition of colonoscopy 10 years after the index examination is recommended. The 2020 ESGE also recommends surveillance colonoscopy after 3 years for patients with complete removal of any serrated polyp ≥10 mm or with dysplasia. Second, patients with positive margins require further radiological and endoscopic surveillance. Third, serrated lesions with dysplasia are also recommended for further follow-up with a short interval.

Given that LAMN is associated with risk of peritoneal dissemination, the differential diagnosis between LAMN and serrated lesions is very important. A major achievement was made by consensus-based histopathologic classifications on behalf of the PSOGI regarding appendiceal mucinous neoplasms (AMNs). According to the PSOGI criteria, LAMN is defined as low-grade cytologic atypia and without invasive infiltration, with one of the following features, including loss of muscularis mucosae, fibrosis of submucosa, pushing invasion, dissection of acellular mucin in the appendiceal wall, undulating or flattened epithelial growth, rupture of the appendix, and mucin and/or cells outside the appendix (4). Due to the heterogeneity of tumors, there may be some morphological overlaps between LAMNs and serrated lesions, and LAMNs may have a region mimicking a serrated architecture. Some SLDs also demonstrate focal atypical hyperplasia of flattened or scalloped epithelium, resembling LAMNs, as shown in our cases. The key criteria of differential diagnosis are the integrity of lamina propria and the presence of dysplastic mucinous epithelium. Our study suggests that loss of lamina propria, replacement with dysplastic mucinous epithelium, and mural fibrosis may be classified as LAMN. However, the establishment of all these features must be based on the complete evaluation of the appendix. Moreover, the entirely submitted appendix is essential for identifying mucinous deposits that cannot be recognized by the naked eyes and for clarifying tumor stage. For LAMN pT3 and pT4, clinical follow-up with periodic abdominal and pelvic imaging for 10 years is recommended (17).

Appendiceal diverticula are classically divided into acquired and congenital types. The majority of diverticula are acquired, with herniation of appendiceal mucosa and submucosa through microanatomical defects in the muscularis propria (18). The incidence ranged between 0.004% and 2.1% from appendicectomy studies (19), but the actual incidence may be higher, because it may be overlooked in macroscopic examination for almost two-thirds of the cases without grossly visible changes. The present study suggests that thorough examination could improve detection rate up to 3.8%. Pasaoglu et al. also revealed that the prevalence of diverticulum was 4.8% due to careful macroscopic samplings (20).

ADD is usually an incidental finding and clinically asymptomatic. When symptomatic, it is usually complicated by acute or chronic diverticulitis with or without acute appendicitis (19). Our study showed that more than half of the patients presented with acute appendicitis. Diverticula have a high rate of perforation compared with acute appendicitis and the perforation rate is up to 30% (21). Ruptured appendiceal diverticula lead to mucin deposits within the subserosa and mesoappendix and on the visceral peritoneal surface, which may cause diagnostic confusion of LAMN. ADD can also manifest as mucosal atrophy caused by intraluminal pressure, resulting in the reduction or even disappearance of crypts. Residual flattened surface epithelium of the atrophy mucosa may also raise concerns about flattened dysplastic epithelium of LAMN. Two cases of ruptured appendiceal diverticula in group 1 were initially misdiagnosed as appendiceal mucinous neoplasms, and similar misdiagnosis also occurred in previous studies (18, 22). One misdiagnosed case was completely submitted due to suspicious macroscopic observations, and the other was resampled due to microscopic findings. Therefore, the submission of a complete appendix is the prerequisite to identify these lesions. It is also necessary to raise the awareness of differential diagnosis. Several clues may be helpful for accurate identification. First of all, half of diverticula had multiple locations, particularly 64.3% of the entire appendix submission group. Continuity between the diverticula and appendiceal lumen is not always seen on the initial sections of the appendix (17). Careful examination of other sections of the entirely submitted appendix and looking for the intact diverticulum in the background would increase the diagnostic confidence of ADD. Other key morphological features that can distinguish ADD from LAMN include mucosal structures with lamina propria, crypt architecture, and nuclear abnormalities. Lowes et al. described a series of 74 appendiceal diverticula and found that non-neoplastic crypts, preserved mucosal architecture with lamina propria, and a lack of nuclear abnormalities were significantly associated with ADD, while loss of lamina propria, a filiform architecture, and hypermucinosis were regarded as important features of LAMN (18).

It should be noted that ADD can be combined with serrated lesions, which challenges the PSOGI criteria and makes the diagnosis even more difficult. ADD may cause eversion of appendiceal serrated lesions on the serosal surface. It is unclear whether this biological behavior would lead to intraperitoneal dissemination, which requires long-term clinical and radiological follow-up.

In conclusion, the present study suggests that appendiceal serrated lesions, diverticula, and some LAMNs may be underestimated due to insufficient routine sampling. More importantly, thorough examination and evaluation are essential to distinguish the key pathological features between entities with different biological behaviors and prognosis. Evaluating the entire part of each appendix will lead to an increase in economic costs and daily workload, but underdiagnosis or misdiagnosis may cause significant medical, social, psychological, ethical, and legal issues. Therefore, we emphasize the importance and necessity of careful gross assessment and histologic examination of the entire appendectomy specimen.

## Data Availability Statement

The raw data supporting the conclusions of this article will be made available by the authors, without undue reservation.

## Ethics Statement

The studies involving human participants were reviewed and approved by the Ethics Committee of the Aerospace Center Hospital, Beijing, China. Written informed consent to participate in this study was provided by the legal guardian/next of kin of the participants.

## Author Contributions

The author contributions were as follows: FL and CQ conceived and designed the project. FL, YL, FH, RM, and DW collected and assembled the data. FL and YL performed the analysis and interpretation. FL and CQ contributed to the draft of the manuscript. All authors contributed to manuscript revision and have read and approved the submitted version.

## Funding

This research was supported by the Science Foundation of Aerospace Center Hospital (YN201915).

## Conflict of Interest

The authors declare that the research was conducted in the absence of any commercial or financial relationships that could be construed as a potential conflict of interest.

## Publisher’s Note

All claims expressed in this article are solely those of the authors and do not necessarily represent those of their affiliated organizations, or those of the publisher, the editors and the reviewers. Any product that may be evaluated in this article, or claim that may be made by its manufacturer, is not guaranteed or endorsed by the publisher.

## References

[1] Misdraji J Carr NJ Pai RK. Appendiceal Serrated Lesions and Polyps. Appendiceal Mucinous Neoplasm. In: WHO Classifcation of Tumours of Digestive System. Lyon, France: IARC Press (2019). p. 141–6.

[2] Sato K Banshodani M Nishihara M Nambu J Kawaguchi Y Shimamoto F. Sessile Serrated Adenoma/Polyp Leading to Acute Appendicitis With Multiple Pyogenic Liver Abscesses: A Case Report. Int J Surg Case Rep (2018) 42:38–43. doi: 10.1016/j.ijscr.2017.11.057 29216529 PMC5725155

[3] Casella C Villanacci V Ubiali A. Acute Appendicitis Associated to “Serrated” Adenoma: A Case Report. Ann Ital Chir (2004) 75(6):691–5. doi: 15960366 15960366

[4] Carr NJ Cecil TD Mohamed F Sobin LH Sugarbaker PH Gonzalez-Moreno S. A Consensus for Classification and Pathologic Reporting of Pseudomyxoma Peritonei and Associated Appendiceal Neoplasia: The Results of the Peritoneal Surface Oncology Group International (PSOGI) Modified Delphi Process. Am J Surg Pathol (2016) 40(1):14–26. doi: 10.1097/PAS.0000000000000535 26492181

[5] Yantiss RK Panczykowski A Misdraji J Hahn HP Odze RD Rennert H. A Comprehensive Study of Nondysplastic and Dysplastic Serrated Polyps of the Vermiform Appendix. Am J Surg Pathol (2007) 31(11):1742–53. doi: 10.1097/PAS.0b013e31806bee6d 18059232

[6] Pai RK Mákinen MJ Rosty C. Colorectal Serrated Lesions and Polyps. Tumours of the Colon and Rectum. In: WHO Classifcation of Tumours of Digestive System. Lyon, France: IARC Press (2019). p. 163–9.

[7] Pai RK Hartman DJ Gonzalo DH Lai KK Downs-Kelly E Goldblum JR. Serrated Lesions of the Appendix Frequently Harbor KRAS Mutations and Not BRAF Mutations Indicating a Distinctly Different Serrated Neoplastic Pathway in the Appendix. Hum Pathol (2014) 45(2):227–35. doi: 10.1016/j.humpath.2013.10.021S0046-8177(13)00452-8[pii 24439221

[8] Renshaw AA Kish R Gould EW. Sessile Serrated Adenoma is Associated With Acute Appendicitis in Patients 30 Years or Older. Am J Clin Pathol (2006) 126(6):875–7. doi: 10.1309/BF5KLH7J547AXAA0 17074693

[9] Yuyucu Karabulut Y Savas B Kursun N Ensar A. Serrated Lesions of the Appendix: Do They Differ From Their Colorectal Counterparts? Turk J Gastroenterol (2014) 25(1):29–34. doi: 10.5152/tjg.2014.4056 24918127

[10] De Palma FDE D’Argenio V Pol J Kroemer G Maiuri MC Salvatore F. The Molecular Hallmarks of the Serrated Pathway in Colorectal Cancer. Cancers (Basel) (2019) 11(7):1017. doi: 10.3390/cancers11071017 PMC6678087 31330830

[11] Pai RK Bettington M Srivastava A Rosty C. An Update on the Morphology and Molecular Pathology of Serrated Colorectal Polyps and Associated Carcinomas. Mod Pathol (2019) 32(10):1390–415. doi: 10.1038/s41379-019-0280-2 31028362

[12] Anderson JC. Pathogenesis and Management of Serrated Polyps: Current Status and Future Directions. Gut Liver (2014) 8(6):582–9. doi: 10.5009/gnl14248 PMC4215442 25368744

[13] Alakus H Babicky ML Ghosh P Yost S Jepsen K Dai Y. Genome-Wide Mutational Landscape of Mucinous Carcinomatosis Peritonei of Appendiceal Origin. Genome Med (2014) 6(5):43. doi: 10.1186/gm559 24944587 PMC4062050

[14] Munari G Businello G Mattiolo P Pennelli G Sbaraglia M Borga C. Molecular Profiling of Appendiceal Serrated Lesions, Polyps and Mucinous Neoplasms: A Single-Centre Experience. J Cancer Res Clin Oncol (2021) 147(7):1897–904. doi: 10.1007/s00432-021-03589-4 PMC8164605 33712927

[15] Yanai Y Saito T Hayashi T Akazawa Y Yatagai N Tsuyama S. Molecular and Clinicopathological Features of Appendiceal Mucinous Neoplasms. Virchows Arch (2021) 478(3):413–26. doi: 10.1007/s00428-020-02906-5 32821969

[16] Hassan C Antonelli G Dumonceau JM Regula J Bretthauer M Chaussade S. Post-Polypectomy Colonoscopy Surveillance: European Society of Gastrointestinal Endoscopy (ESGE) Guideline - Update 2020. Endoscopy (2020) 52(8):687–700. doi: 10.1055/a-1185-3109 32572858

[17] Valasek MA Pai RK. An Update on the Diagnosis, Grading, and Staging of Appendiceal Mucinous Neoplasms. Adv Anat Pathol (2018) 25(1):38–60. doi: 10.1097/PAP.0000000000000178 29016471

[18] Lowes H Rowaiye B Carr NJ Shepherd NA. Complicated Appendiceal Diverticulosis Versus Low-Grade Appendiceal Mucinous Neoplasms: A Major Diagnostic Dilemma. Histopathology (2019) 75(4):478–85. doi: 10.1111/his.13931 31166613

[19] Abdullgaffar B. Diverticulosis and Diverticulitis of the Appendix. Int J Surg Pathol (2009) 17(3):231–7. doi: 10.1177/1066896909332728 19233860

[20] Pasaoglu E Leblebici C Okcu O Boyaci C Dursun N Hande Yardimci A. The Relationship Between Diverticula and Low-Grade Mucinous Neoplasm of the Appendix. Does the Diverticulum Play a Role in the Development of Periappendicular Mucin Deposition or Pseudomyxoma Peritonei? Pol J Pathol (2016) 67(4):376–83. doi: 10.5114/pjp.2016.62829 28547966

[21] Yamana I Kawamoto S Inada K Nagao S Yoshida T Yamashita Y. Clinical Characteristics of 12 Cases of Appendiceal Diverticulitis: A Comparison With 378 Cases of Acute Appendicitis. Surg Today (2012) 42(4):363–7. doi: 10.1007/s00595-012-0152-6 22358430

[22] Hsu M Young RH Misdraji J. Ruptured Appendiceal Diverticula Mimicking Low-Grade Appendiceal Mucinous Neoplasms. Am J Surg Pathol (2009) 33(10):1515–21. doi: 10.1097/PAS.0b013e3181abe31b 19623035

